# Light Transmission of Various Aesthetic Posts at Different Depths and Its Effect on Push-Out Bond Strength, Microhardness of Luting Cement

**DOI:** 10.3390/medicina58010075

**Published:** 2022-01-04

**Authors:** Satheesh B. Haralur, Turki Abdullah Alasmari, Mohammed Hussin Alasmari, Hafiz Mohammed Hakami

**Affiliations:** 1 Department of Prosthodontics, College of Dentistry, King Khalid University, Abha 62529, Saudi Arabia; 2 College of Dentistry, King Khalid University, Abha 62529, Saudi Arabia; turkimosafiq@gmail.com (T.A.A.); dr.Mohd.asmri@gmail.com (M.H.A.); Hmmh450@gmail.com (H.M.H.)

**Keywords:** fiber-reinforced composite post, zirconia ceramic post, highly translucent zirconia, light translucency, microhardness, push-out bond strength

## Abstract

*Background and Objectives*: One requirement for the cemented post is the light transmittance on its entire length up to the deepest portion of a root canal to ensure the complete polymerization of resin cement. This study aimed to determine the light transmission ability in different aesthetic posts at different depths and its effect on the push-out bond strength and microhardness of luting cement at the corresponding interface. *Materials and Methods*: Twenty endodontic posts from glass fiber posts (GFP), zirconia ceramic posts (ZCP), and highly translucent zirconium oxide posts (HTZP) were sequentially sectioned into 12.8 and 4 mm lengths after recording the light intensity using a dental radiometer. Sixty single rooted premolar teeth root canals were treated and implanted vertically in a resin block. The post space was prepared and cemented with GFP, ZCP, and HTZP posts with twenty samples each. The root portion of teeth samples were sectioned into cervical, middle, and apical portion. A universal testing machine was utilized for the push-out bond strength test for the first ten samples from each group. The remaining ten samples from each group were used for the microhardness test using a micro-indenter instrument. The data were statistically analyzed using one-way Analysis of variance and Tukey HSD tests at *p* < 0.05. *Results*: The GFP endodontic postpresented with significant highest light translucency compared to HTZP, which was significantly higher than ZCP. GFP posts showed significantly higher bond strength per unit area compared to ZCP at analogous cross sections. The hardness of luting cement was also significantly higher amongst all tested endodontic posts. *Conclusions*: GFP high light translucency enhanced the curing of the luting resin cement that resulted in harder cement and a stronger bond supported by hardness and push-out tests. These findings suggest that GFP is preferred to be used with light-cured luting cements for restoration of endodontically treated teeth.

## 1. Introduction

A contemporary society driven by social media has enhanced the demand for aesthetic treatments to improve physical appearance [1]. Dental appearance plays a significant role in facial aesthetics [2]. Hence, dental professionals are in constant pursuit of advancing the treatment methods and materials to achieve improved aesthetic outcomes. All-ceramic indirect restorations are widely employed in modern dental practice to accomplish satisfactory functional and aesthetic results [3]. Endodontically treated teeth with insufficient tooth structure often need a post-core as a foundation for final indirect restoration. Cast metal post-core on the anterior teeth may lead to compromised aesthetics due to shine through the semi-translucent all-ceramic crown or thin gingival tissue [4]. Moreover, posts from nonprecious alloys could lead to the leaching of corrosion products and discoloration of the soft tissue–ceramic interface. Hence, complementary aesthetic endodontic posts are advocated while restoring teeth with all-ceramic restorations. 

Besides high tensile and fatigue strength, the compatible modulus of elasticity with radicular dentin is considered as a primary attribute that inhibits catastrophic root fracture [5]. The fiber-reinforced composite (FRC) is routinely used because of its favorable modulus of elasticity, easy clinical procedure, retrievability, and aesthetic color. The internal shade of post-core material affects the final shade of all-ceramic restoration. Zirconia ceramic post-core, because of their dentin-like shade, diffusion, and absorption of the incident light, increases the depth of translucency and gives a natural appearance to the final all-ceramic restorations [6]. 

Post dislodgement and root fracture are the most common causes for failure of post-restored endodontically treated teeth. Debonded loose posts within the root canal act as a wedge, resulting in a high concentration of force and consequent root fracture [7]. Hence, a strong and durable bond between the post–radicular dentin interface is crucial for the long-term success of restored teeth. Compromised post bond strength is attributed to multiple factors such as unfavorable root canal morphology, polymerization shrinkage stress of luting cement [8], and high C-factor [9]. Researchers report the direct relation between polymerization of the resin-luting cement and its bond strength to dentin [10]. The ability to transmit the light from the photo polymerizing unit to the deepest radicular section is vital for the complete polymerization of resin luting cement. Previous studies reported that light transmission through the post is affected by material, physical composition, and optical properties [11]. Resin-based cements are preferred as luting cement due to compatible modulus elasticity, favorable stress distribution, and optimum retention [12]. Clinicians prefer dual-cured cement because of its extended working time, and high degree of conversion in the absence of adequate light [13]. However, dual-cure resin cement is reliant on light activation [14]. Hence, a compromised degree of conversion is expected with the lack of an adequate light source.

The light transmission at different depths of the root canal is crucial for optimum polymerization and performance of resin luting cement. The incomplete conversion at the deeper radicular section leads to poor bond strength and reduced mechanical properties of luting cement. There is a paucity of dental literature regarding the light transmission capability of the different aesthetic posts at various depths, their effect on bonding performance, and the mechanical properties of resin luting cement. This present study evaluated the light transmission ability of FRC, zirconia ceramic posts, and high translucency zirconia posts at various depths. The aim of the study also included the assessment of push-out bond strength and microhardness of luting cement at different radicular sections. The hypotheses of the study were: (1) that depth or post materials would not influence light transmission; (2) that the bond strength per unit surface area and microhardness of luting cement at different radicular sections would be similar.

## 2. Materials and Methods

Institutional ethical review board approval was obtained for study protocol (IRB/KKUCOD/ETH/2020-21/066). Sixty single-rooted human premolar teeth were collected from oral surgery clinics. The teeth included in the study were extracted for therapeutic purposes such as orthodontics or periodontal reasons. The mean root length of the teeth was 15.81 ± 1.72 mm. Root canal morphology was validated with mesial–distal and buccolingual periapical radiographs. The exclusion criteria included caries, previous restoration, root canal treatment, crack and fractures, and anomalies. Teeth samples were cleared from remnant periodontal tissues with an ultrasonic scaler, subsequently immersed for two weeks in 10% formalin solution for disinfection. The teeth were stored in distilled water at room temperature and used within 60 days after extraction.

**Figure 1:**
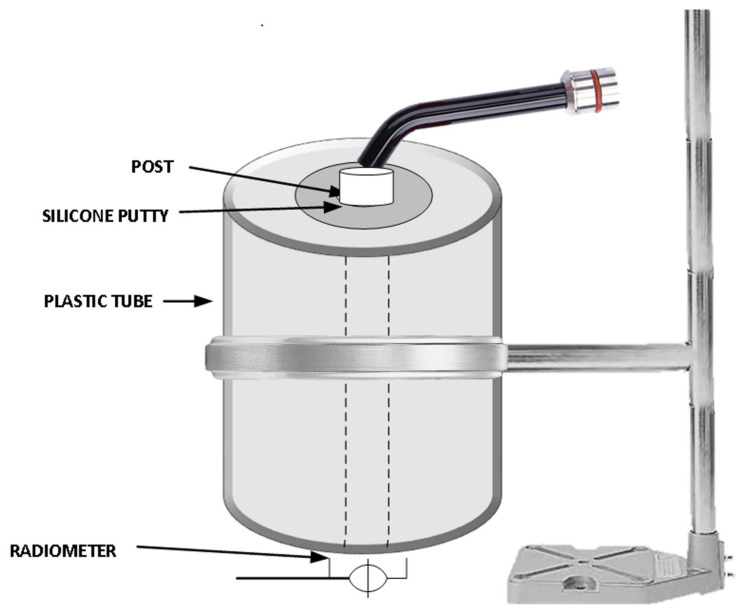
Schematic illustration of the Light transmission measurement process.

Ten endodontic posts from glass fiber tapered posts (GFP,1.6 mm, RelyX fiber post, 3M Oral Care, St. Paul, MN, USA), parallel conical-shaped zirconia-based ceramic posts (ZCP, 1.7 mm, Cosmo post, Ivoclar Vivadent AG, Schaan, Liechtenstein), and tapered custom-milled highly translucent zirconium oxide posts (HTZP,1.6 mm Zolid ht+ white, Amann Girrbach AG, Koblach, Austria) were utilized in the study. Autopolymerizing clear polymethyl methacrylate acrylic resin mixed with black pigment was poured into a 10 × 25 mm hollow plastic tube. Each post was coated with petroleum jelly (Vaseline, Unilver, Dubai, UAE) and was implanted vertically with the help of a dental surveyor into the acrylic resin. Post-polymerization of acrylic resin, the post was sectioned, leaving 2 mm protruding above the surface of acrylic resin and the post implanted until it come into contact with the bottom of the tube. As the post was lubricated with petroleum jelly, it facilitated the removal of post and acrylic mold with the standardized height and an internal orifice was fabricated for testing the light translucency. The standardized diameter of the internal orifice was 1.8 mm at the top and 1.5 mm at the bottom. An acrylic mold at the height of 8 and 4 mm was also fabricated following a similar protocol. A custom-made metal jig was fabricated to maintain the position of acrylic mold and light source over the radiometer. Silicone impression material was used to seal the space between the post and internal orifice. The post was inserted within the orifice, and an acrylic cylinder was placed vertically over the radiometer sensor area (Bluephase Meter II, Ivoclar Vivadent AG, Schaan, Liechtenstein). The light source (Elipar S10, 3M ESPE, St. Paul, MN, USA) was placed over the tip of post for 10 seconds; the transmitted light power was registered at the bottom surface in the dental radiometer (Figure 1). The dental radiometer used in the study had a measuring range for wavelength ranges of 380–550 nm and light intensities of 300–12,000 mW/cm^2^. The light source had the intensity at 1200 mW/cm² and a utilizable wavelength range between 430–480 nm. Mean irradiance power was recorded three times for each sample to avoid the errors and an average was calculated as the light transmission value. After recording the light transmission at a 12-mm length, the post was sequentially sectioned to 8- and 4-mm height and light transmission was recorded with the help of corresponding acrylic mold. Light intensity less than 300 mW/cm^2^ was not able to be recorded by radiometer. The post samples in which no light intensity was recorded by radiometer were assigned an estimated value of 270 mW/cm^2^ for statistical purposes. 

### 2.1. Microhardness and Push-Out Bond Strength Test

**Figure 2:**
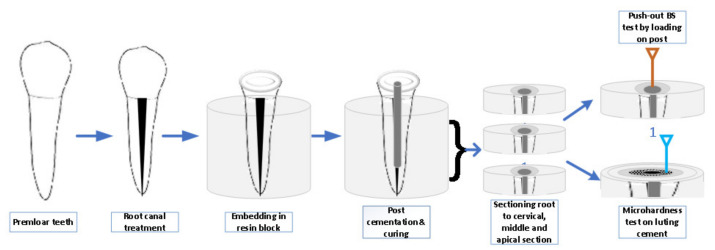
Push-out test and microhardness methodology flowchart.

All the teeth samples were root canal-treated with a rotary endodontic instrument (Protaper next, Dentsply Sirona, Charlotte, NC, USA) up to ×3 file, obturated with a corresponding size gutta-percha cone using a eugenol-free sealer (Roeko, Coltane Gmbh co, Langenau, Germany). Later, they were sectioned at a cementum–enamel junction with a double-sided diamond disc (Kerr Corporation, Orange, CA, USA), implanted vertically within acrylic resin with a dental surveyor. The standardized post space width (according to post width) and 13-mm depth was established by calibrated post drill (Fibrekleer, Pentron clinical, Orange, CA, USA) (Figure 2). Twenty randomly selected teeth samples were cemented each from GFP, ZCP, and HTZP post groups. All endodontic posts were cemented with dual-cure adhesive resin luting cement (RelyX ARC, 3M ESPE, St. Paul, MN, USA) and light-cured (Elipar S10, 3M ESPE, St. Paul, MN, USA) for 40 seconds. The light was guided vertically along the length of the post. The optical power of e 1200 mW/cm² intensity was maintained constantly during the experiment.

**Figure 3:**
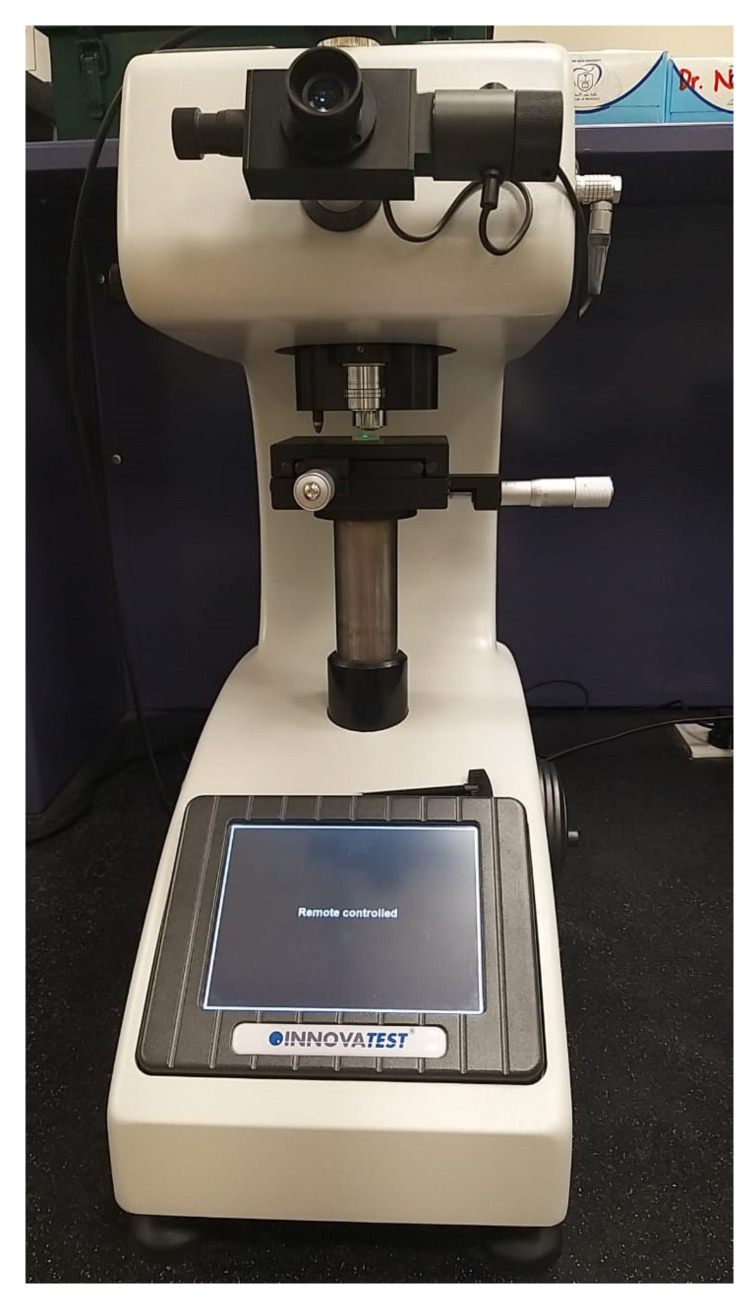
Microhardness testing on the teeth sample.

**Figure 4:**
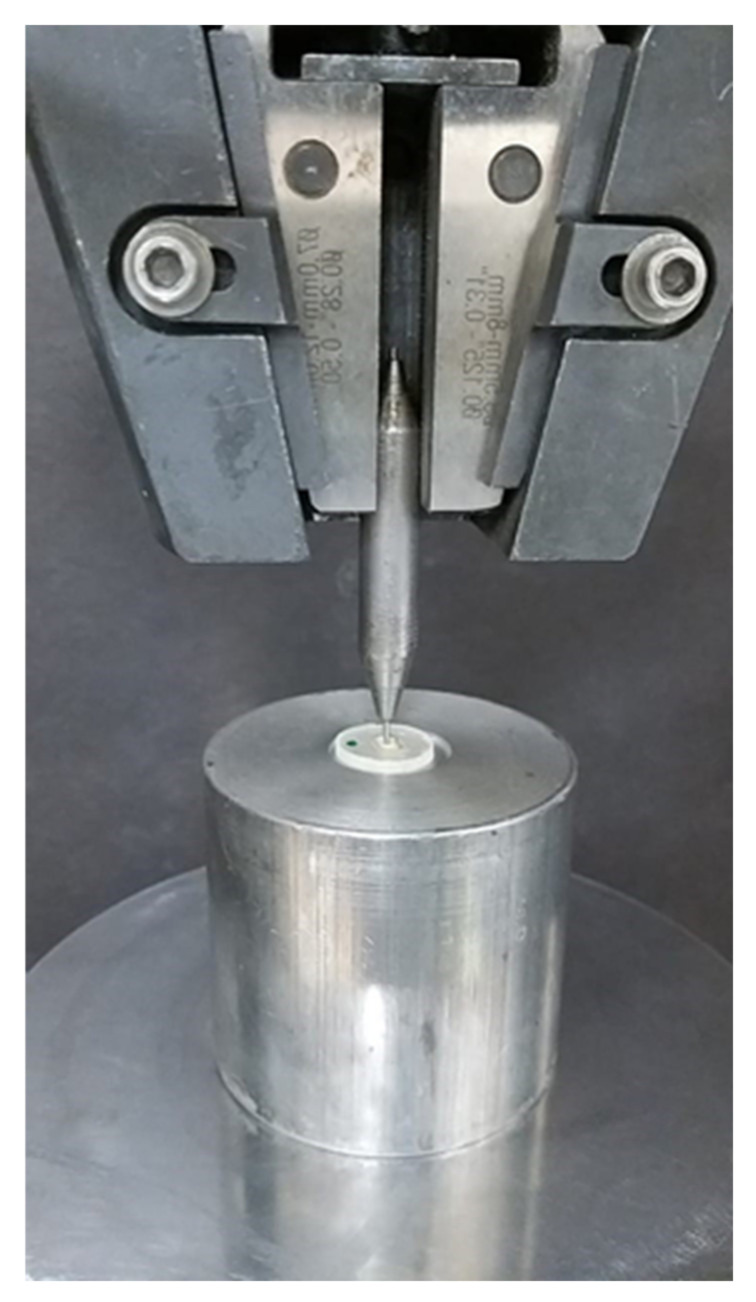
Push-out bond strength testing on universal testing machine.

Ten teeth samples from each post group were utilized for the microhardness test. A coronal 1-mm teeth slice was sectioned and discarded to eliminate the effect of excess luting cement over tooth substrate. The teeth samples were sectioned at coronal, middle, and apical third with 4-mm thickness. The micro-indenter instrument (FALCON-500, INNOVATEST Europe BV, Borgharenweg, Maastricht, NL, Canada) was used to test the microhardness of luting cement in (Figure 3) each cross section. The microhardness test was performed over resin luting cement using Vickers digital micro-indenter, applying 200 gf with a retention time of 15 s. Each sample was tested at 3 different locations and an average value was expressed as the Vickers hardness number (VHN). The remaining ten samples from each group were subjected to 1500 thermocycling (1100; SD Mechatronik, Feldkirchen–Westerham, Germany) between 5 and 55 °C with the dwell time of 30 s and transfer time of 10 s. Subsequently, the teeth samples were sectioned at the coronal, middle, and apical third. Two-mm slices from each cross section were utilized for the push-out bond strength. The sectioned samples with coronal parts facing down were placed over a universal testing machine (Instron, Norwood, MA, USA), and an apically directed load from one-mm diameter plunger at 0.5 mm/min speed was applied on the post (Figure 4). The force was applied until the post dislodged from a root canal and the maximum load was recorded.

The bonding area was calculated using the formula in Equation (1) and push-out bond strength results were expressed in MPa per each calculated unit area.

π(R_(1 )+ R_2)√(h^2+〖(R_(1 )- R_2)〗^2 )(1)

The π is constant at 3.14. R1 and R2 are the post radii at a larger and smaller radius. The ‘h’ is the thickness of the post. A digital caliper (fino Pra Ceci caliper; FINO GmbH, Bad Bocklet, Germany) was used to measure the teeth sample dimensions.

### 2.2. Statistical Analysis

The statistical analysis was performed using SPSS 19 software (IBM Corporation, Armonk, New York, NY, USA). Data were analyzed by one-way ANOVA and Tukey HSD tests. The level of statistical significance was determined at *p* < 0.05. 

## 3. Results

**Figure 5:**
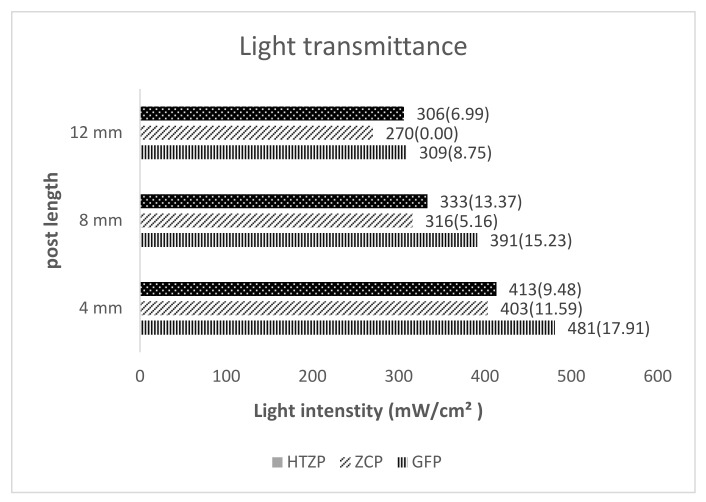
Mean light intensity (mW/cm²) among the different post at various depth. HTZP: highly translucent zirconium oxide posts; GFP: glass fiber posts; ZCP: zirconia ceramic posts.

The mean light intensity (mW/cm²) among the different posts at various lengths is summarized in Figure 5. The highest light transmission intensities of 481(17.91), 391(15.23), and 309(8.75) mW/cm² was observed in the GFP post at experimental post lengths of 12, 8, and 4 mm. The HTTP post followed this with 413(9.48), 333(13.37), and 306(6.99) mW/cm² at corresponding lengths. The zirconia ceramic post groups with 403(11.59), 316(5.16), and 270(0.00) mW/cm² at 12-, 8-, and 4-mm post lengths, respectively, recorded the lowest mean light translucency. All ZCP samples with 12-mm length had light intensity lesser than 300 mW/cm^2^.

**Figure 6:**
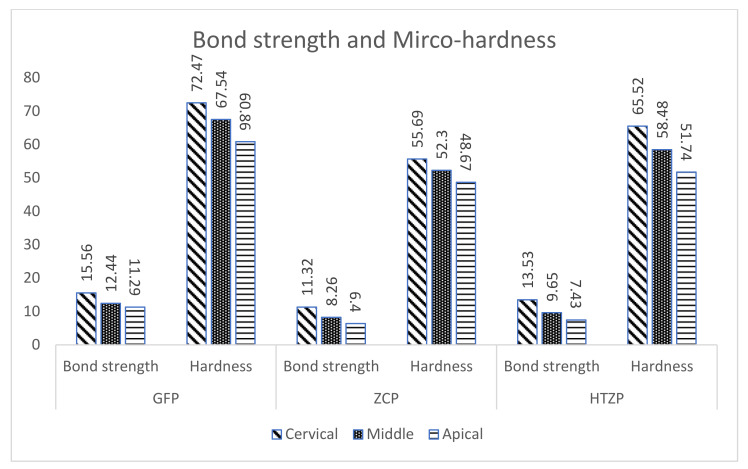
Mean and standard deviations (MPa) of push-out bond strength and hardness (HV) of luting cement.

Push-out bond strength was greater at the cervical cross section compared with the middle and apical cross section across all the rested groups (Figure 6). GFP posts presented push-out bond strength at 15.56(0.32) (cervical), 12.44(0.52) (middle), and 11.29(0.34) Mpa (apical) compared to 11.32(0.35), 8.26(0.36), and 6.40(0.33) Mpa at an analogous cross section from ZCP. Moreover, the hardness of luting cement was also higher amongst GFP posts with 72.47(0.86), 67.54(0.74), and 60.46(0.83) HV at the cervical, middle, and apical cross sections, respectively. The luting cement hardness for HTZP posts at the cervical, middle, and apical cross sections were 65.52(1.36), 58.48(1.11), and 51.74(0.73) HV. The corresponding values for the zirconia ceramic posts were 55.69, 52.30, and 48.67, respectively.

A one-way ANOVA () was conducted to compare the effect of different cross sections on the push-out bond strength of endodontic post fabricated from different aesthetic post materials. One-way ANOVA analysis demonstrated the effect of different post material on the push-out bond strength was significant in the coronal cross section, F (2.27) = 341.041, *p* = 0.000. Analogous significant difference in push-out bond strength was observed in middle cross section, F (2.27) = 184.617, *p* = 0.000 and apical cross section, F (2.27) = 790.289, *p* = 0.000. The Shapiro–Wilk test for normality showed the normal data distribution with *p* > 0.05 in various aesthetic post groups at different cross sections. Tukey HSD post hoc multiple comparison tests () showed the significant difference between push-out bond strength of all endodontic post materials at different cross sections.

One-way ANOVA analysis showed the effect of different post materials on the hardness of luting cement was significant at cervical region F (2.27) = 678.036, *p* = 0.000; middle cross section F (2.27) = 721.691, p = 0.000; and apical section F (2.27) = 642.655, *p* = 0.000. Tukey HSD post hoc multiple comparison tests () also showed the significant difference between the hardness of luting cement of all endodontic post materials at different cross sections.

## 4. Discussion

Increased demand for all-ceramic indirect restorations has led to the concurrent utilization of aesthetic-colored endodontic posts. Besides the modulus of elasticity and biomechanical properties, satisfactory curing of luting cement and dental adhesives are reported to play a significant role in long-term success [15]. To develop adequate bond strength and mechanical properties, luting cement requires ample light sources to achieve polymerization. Incompletely polymerized resin could leak through the apical seal and lead to inflammatory, cytotoxic, and mutagenic reactions of periapical and periodontal tissues [16]. Root canal morphology presents greater challenges for light polymerization of the luting cement. Hence, light transmission and scattering attributes of the endodontic post are crucial for a higher degree of conversion of luting cement at the apical region of a root canal.

The results of all the evaluated posts showed the attenuation of light intensity at deeper post length. Hence, the null hypothesis that the depth of post would not affect the light transmission was rejected. Previous researchers showed a significant reduction in the quality of light transmission with increased depth [17]. The significant reduction in transmitted light amounts, especially at the middle and apical end of the post, was also reported by Moazzami et al. [18]. The reduction in light transmission with increased depth was linear in all the tested groups. These results are consistent with those of Vieira et al. [19], who observed a significant reduction in light transmission power from cervical to apical third. This reduction of light transmission with increased post length seems to follow the Lambert–Beer Law, which explains that light absorption and dispersion reduce the energy density in an exponential form with a negative exponent in resin materials [20]. The intensity of the light-curing unit according to the ISO standards is 300 mW/cm^2^ with wavelengths of 400–515 nm [21]. However, researchers have reported that the minimum radiant exposure and irradiance to trigger an adequate polymerization of the light-cured resin cement were 6 J/cm^2^ and 100 mW/cm^2^, respectively [22]. The light intensity at 200 mW/cm^2^ resulted in adequate curing and degree of conversion of luting cement. Rueggeberg et al. [23] recommend that sources with intensity values less than 233 mW/cm^2^ should not be used because of their poor cure characteristics.

The light intensity (mW/cm²) was recorded to be significantly the highest in GFP post at all assessed lengths of 12, 8, and 4 mm, followed by HTZP and ceramic zirconia posts. The type, size, filler concentration, and pigment color affect the quality of light absorption, reflection, and transmission in the post [19,24]. Therefore, the difference in the light transmission values of posts evaluated in the study could be attributed to the chemical and structural differences between them. Soares et al. [25] found among the post systems, ceramic posts showed the highest radiodensity level, followed by a metallic post, carbon fiber posts, glass fiber posts, and the carbon fiber post covered with quartz fiber. GFP posts comprise a cross-linked polymer matrix, wherein fiber resembles the core, and the matrix assumes the role of cladding. Once the light irradiated GFP posts were comparable to multi-mode fiber optic. The light radiations were conducted along with the fiber core with total internal reflection. The critical angle is the angle determined by the refractory indices of fiber and matrix cladding. If the angle of light incidence is greater than the critical angle, the refracted ray is reflected back into the fiber core. Consequently, the light is conveyed onward with the fiber. The variability in fiber orientation, diameter, matrix, and filler results in different refraction indexes, affecting light conduction along with the post [16]. The ceramic zirconia post had lesser light transmission values, possibly because the presence of silica zirconia adversely affected the light-transmitting properties. The results agreed with the findings of Morgan et al. [26]. They reported the lesser light transmission through ceramic zirconia posts compared to fiber posts.

The study demonstrated that high translucent zirconia has a significantly higher light transmission than conventional ceramic-zirconia posts. The conventional opaque zirconia made more translucent by reducing high refractory alumina content from 0.25% to 0.05% [27]. Recently, the improvement in translucency has been accomplished by stabilizing cubic-phase crystal at room temperature via increasing yttria contents (4–5 mol%) from conventional tetragonal zirconia polycrystal with 3 mol% yttria [28]. The cubic-phase system is optically isotropic, and consequently has an equal refractory index in all directions of the crystal lattice. Moreover, it has a lesser grain boundary area due to its grain size beyond the red portion of the visible wavelength [29]. With all the earlier mentioned modifications in content, the structural matrix has significantly enhanced light transmittance monolithic zirconia [30].

Rueggeberg FA et al. [31] reported that light intensity and exposure duration influences the polymerization of light cure resins. Results of the present study indicate the endodontic post with better light transmission had luting with high microhardness. The microhardness of luting cement was progressively reduced from cervical to apical cross section within the same group. We could attribute it to lesser light transmission over the longer length from a light source [32]. These findings were consistent with the findings of e Silva et al. [33], who reported that the superficial depth presented a higher degree of conversion values than those in the medium and deep depths. Hence, researchers proposed using a light-transmitting post to facilitate the resin cure depth at the apical region [34,35]. The higher intensity light enables more photons availability for absorption by photosensitizers, resulting in more free radicals to initiate and propagate the polymerization process [36].

Stylianou et al. [37] reported associations of polymerization depth and weight of resin with the type of post regarding different composition, density, and optical properties. The decrease in the degree of conversion relative to the depth depended on the light transmission capacity of the posts was also corroborated by Kim et al. [38]. A reduced degree of conversion diminishes the mechanical properties of luting resin, including hardness and fracture resistance [39]. The difference in microhardness of dual-cure resin cement confirmed that high opacity post prevents light propagation. Silva Junior [40] reported that higher translucent posts promoted greater microhardness values at the apical root levels. Lower microhardness of resin at apical section compared to middle and cervical third was also reported by Ceballos et al. [41] and Ozcan et al. [42].

Bond strengths reduced significantly from coronal to apical root canal regions across all the tested groups. Wang et al. [43] reported similar results of higher bond strength at the coronal region. Dual-cure resin cement has been shown to be reliant on the self-cure mechanism in the absence of adequate light activation [44]. Research reports show the compromised polymerization efficiency in self-cure mode [45]. Hence, the resin cement at the apical and middle sections is mostly dependent on self-polymerization than physical cure compromising bond strength. Reginato et al. [46] confirmed the higher bond strength of the translucent post and greater bond strength at the cervical third than the middle and apical third. Szesz et al. [47] observed that higher radiant exposure values increase the bond strength of adhesive systems; presumably cervical third received the greatest radiant exposure. Researchers reported only 19.06% and 8.37% of emitted light is transmitted to the middle and apical third [48]. However, our results contradict the finding of Quitero et al. [49] who reported no correlation between post translucency and tensile bond strength of resin luting cement. These include the progressively lesser distribution and densities of dentinal tubules towards the apical region [50] also compromise the bond strength at the apical region. The calculation of bonded surface area is critical while evaluating push-out bond strength. Previous researchers indicated that the shear bond strength of the adhesives depended on the bonded surface area, and showed an inverse association [51,52]. Additional factors such as thicker smear layer, difficulty in access, residual root canal sealer, and gutta-percha jeopardize the push-out bond strength of the root canal at the apical third [53]. Allabban et al. [54]. reported similar results of higher bond strength in all root thirds by glass fiber posts compared to zirconia posts. Alshammary et al. [55], in their evaluation of the bond strength of zirconia post to radicular dentin, demonstrated higher bond strength scores in the cervical area as compared to the apical areas of the root.

Clinical implications of the study results include that clinicians should recognize the significance of delivering enough light energy to the luting cement to achieve the best biomechanical performance. Light transmission capability of posts needs to be considered to determine appropriate light source intensity and exposure time during the cementation procedure.

Limitations of the study include that the study samples were tested at room temperature, whereas intra-oral temperature is comparatively higher at 33 to 35 °C. Higher temperature leads to an increased degree of conversion and polymerization shrinkage of resin cement. Only dual-cure luting cement was evaluated in the study; self-polymerization and light cure luting cement are expected to have varying degrees of conversion. The endodontic post was polymerized immediately after cementation, while a slight delay in curing post cementation is expected in the clinical situation. Hence, due discretion should be observed while extrapolating the results to different adhesive strategies and clinical situations. The present study has not measured the controls especially for light transmittance experiment. Further studies suggested evaluating the different root canal temperatures, various adhesive strategies, and delayed curing on the biomechanical performance of endodontic posts. Given that the luting cement is being cured around the whole circumference of the post, it would be interesting to devise a method to measure light transmission intensities from sides not only along the post. This can be done by creating 4 holes in the side of tubes at angles (0°, 90°, 180°, 270°) at each cross section.

## 5. Conclusions

Within the limitations of this in vitro study, the following conclusions were drawn:

1. The light intensity attenuation with increased depth was observed in all tested posts;
2. The fiber-reinforced composite post showed the highest light transmission intensity at different lengths compared to the high translucent post and ceramic zirconia post;
3. Microhardness of luting cement was consistently higher at cervical cross section across all the posts and recorded highest in the GFP post;
4. Push-out bond strength was correlated with microhardness of luting cement; GFP post recorded the maximum strength at the cervical cross section.

## Data Availability

Data sharing not applicable.
